# Editorial: Integrative analysis for complex disease biomarker discovery

**DOI:** 10.3389/fbioe.2023.1273084

**Published:** 2023-08-21

**Authors:** Hai-Hui Huang, Jie Li, William C. Cho

**Affiliations:** ^1^ Provincial Demonstration Software Institute, Shaoguan University, Shaoguan, China; ^2^ Faculty of Information Technology, Macau University of Science and Technology, Macau, China; ^3^ School of Computer Science and Technology, Harbin Institute of Technology, Harbin, China; ^4^ Department of Clinical Oncology, Queen Elizabeth Hospital, Kowloon, Hong Kong SAR, China

**Keywords:** complex disease, biomarker discovery, integrative analysis, machine learning, network-based analysis, systems-based approaches

Complex diseases are multifactorial and are caused by a combination of genetic, environmental, and lifestyle factors (Ma’ayan et al., 2014). They account for major morbidity and mortality globally, presenting long-standing challenges to researchers and clinicians. In recent years, with the advent of new technologies and analytical methods, we have moved from a low-throughput, single-dimensional approach to studying diseases to a much more integrated, systems-level understanding (Jiang et al., 2022). This shift has opened new avenues for identifying biomarkers for complex diseases, which is crucial for improving disease diagnosis, prognosis, and treatment. The discovery of robust and accurate biomarkers has been made possible through the integration of multi-omics data from different sources. Researchers can extract biomarkers from this multi-omics data and build either a single predictive model based on these biomarkers, multiple models based on different biomarkers combined or use a mixed strategy for integration (Yang et al., 2023).

Examples of successful usage of integrative analysis abound in the field. The Cancer Genome Atlas (TCGA) project, a landmark cancer genomics program, molecularly characterized over 20,000 primary cancer and matched normal samples spanning 33 cancer types (Weinstein et al., 2013). This large-scale project has enabled the discovery of numerous novel cancer genes and biomarkers and has provided an unparalleled resource for understanding cancer biology. Another example is the Alzheimer’s Disease Neuroimaging Initiative (ADNI), which integrates genomic data with imaging and clinical data to identify biomarkers for Alzheimer’s disease (Veitch et al., 2019). Both examples highlight the power of integrative analysis in biomarker discovery and underscore the potential of this approach in the study of complex diseases.

Building on this momentum, the recent Research Topic (RT) “*Integrative analysis for complex disease biomarker discovery*” brings together four compelling studies that exemplify the power of integrative analysis in uncovering new insights into complex diseases. The four selected articles represent a broad cross-section of research on this Research Topic, addressing diverse diseases and methodologies. They encompass a wide range of complex diseases, from fetal growth restriction to drug sensitivity prediction in cancer, joint capsule fibrosis, and breast cancer. These works utilize a range of advanced techniques, including machine learning algorithms, network-based analysis, systems-based approaches, and omics platforms integrated with experimental validation.

The first article, “*An miRNA-mRNA integrative analysis in human placentas and mice: role of the Smad2/miR-155-5p axis in the development of fetal growth restriction*,” by Wu et al., explores the role of microRNA (miRNA) in fetal growth restriction (FGR). The authors performed a miRNA-messenger RNA (mRNA) integrative analysis, which revealed that miR-155-5p was upregulated, and Smad2 was downregulated in fetal-side placental tissues, suggesting a role for the Smad2/miR-155-5p axis in placental pathologies of FGR. This finding contributes to our understanding molecular pathogenesis of FGR, a condition that has broad implications for infant health and development. By identifying the Smad2/miR-155-5p axis as a potential therapeutic target, this study opens up new avenues for the management and treatment of FGR, underscoring the power of integrative analysis in uncovering complex biological systems.

In the second article, “*Prediction of drug sensitivity based on multi-omics data using deep learning and similarity network fusion approaches*,” by Liu and Mei, the authors developed a novel drug sensitivity prediction model using deep learning and similarity network fusion approaches. This model can integrate and analyze multi-omics data, reducing the risk of overfitting and increasing the accuracy of drug sensitivity prediction for targeted and non-specific cancer drugs. The development of this model is a significant step forward in the field of precision oncology. It allows for the prediction of drug sensitivity based on individual patients’ molecular profiles, improving the specificity and efficacy of treatments. Moreover, this work demonstrates the potential of artificial intelligence in biomedical research, particularly in the context of complex disease biomarker discovery.

The third article, “*Platelet-rich plasma attenuates the severity of joint capsule fibrosis following post-traumatic joint contracture in rats*,” by Zhang et al., investigates the antifibrotic potential of platelet-rich plasma (PRP) in the context of joint capsule fibrosis, a condition often accompanying joint trauma and characterized by loss of motion and tissue deformity. The authors found that PRP can attenuate pathological changes of joint capsule fibrosis during PTJC, which may be implemented by inhibiting TGF-β1/Smad2/3 signaling and downstream fibrotic marker expression in joint capsule fibroblasts. This study provides a promising therapeutic strategy for post-traumatic joint contracture, a condition that currently lacks effective treatment options. The use of PRP, an autologous and easily accessible resource, also offers significant advantages over other potential treatments, highlighting the potential of regenerative medicine approaches in managing complex diseases.

Finally, in “*Early myeloid-derived suppressor cells accelerate epithelial-mesenchymal transition by downregulating ARID1A in luminal A breast cancer*,” by Chen et al., the authors demonstrate that early myeloid-derived suppressor cells (eMDSCs) can promote epithelial-mesenchymal transition (EMT) in luminal A breast cancer cells by downregulating ARID1A expression. The study also established a 21-gene signature to predict the infiltration of eMDSCs within breast cancer tissues, which could serve as a valuable predictive biomarker for luminal A breast cancer patients. The findings of this study highlight the critical role of immune cells in the tumor microenvironment and their influence on tumor progression and metastasis. Additionally, the identification of the eMDSCs-ARID1A axis as a potential therapeutic target opens up new possibilities for the treatment of metastatic breast cancer.

The studies within this RT showcase the power of integrative analysis in the discovery of complex disease biomarkers. They demonstrate how multidimensional methods and analyses can enhance our understanding of complex diseases and significantly contribute to the development of novel therapeutic strategies. From the exploration of miRNA-mRNA interactions in fetal growth restriction to the development of a deep learning model for predicting drug sensitivity in cancer, these studies illustrate the potential of integrative analysis in advancing precision medicine. In the era of big data and artificial intelligence, the integration of diverse data types and computational methodologies is becoming increasingly important in biomedical research. The ability to analyze large, multidimensional datasets can shed light on the intricate molecular mechanisms of diseases and facilitate the discovery of robust biomarkers for disease prediction, prognosis, and treatment response. However, the integration of diverse data types and computational methodologies also poses significant challenges. High-dimensional data can lead to overfitting in model development, and the integration of heterogeneous data requires sophisticated statistical and computational methods. Furthermore, the interpretation of results from integrative analyses can be complex and requires careful validation with experimental studies. Despite these challenges, the studies in this RT have shown the significant potential of integrative analysis in the discovery of biomarkers for complex diseases. As demonstrated by these studies, integrative analysis can uncover novel molecular mechanisms, identify potential therapeutic targets, and develop predictive models for disease outcomes.

In conclusion, the integration of computational and experimental approaches is crucial for advancing our understanding of complex diseases and improving patient care. The studies in this RT represent significant contributions to this field, and it is anticipated that the continued development and application of integrative analysis will further accelerate the discovery of biomarkers for complex diseases and advance the field of precision medicine. As the field moves forward, it will be essential to continue developing and refining these methods, as well as validating and implementing the findings in clinical practice.
